# What Helping Babies Breathe knowledge and skills are formidable for healthcare workers?

**DOI:** 10.3389/fped.2022.891266

**Published:** 2023-01-30

**Authors:** Archana B. Patel, Akash Bang, Kunal Kurhe, Savita Bhargav, Patricia L. Hibberd

**Affiliations:** ^1^ Research Unit, Lata Medical Research Foundation, Nagpur, India; ^2^ Department of Medical Research, Datta Meghe Institute of Medical Sciences, Wardha, India; ^3^ Department of Pediatrics, All India Institute of Medical Sciences, Nagpur, India; ^4^ Department of Global Health, Boston University School of Public Health, Boston, MA, USA

**Keywords:** HBB, Helping Babies Breathe, training, neonatal resuscitation, knowledge, skills

## Abstract

**Introduction:**

Most neonatal deaths occur in the first week of life, due to birth asphyxia. Helping Babies Breathe (HBB), is a simulation-based neonatal resuscitation training program to improve knowledge and skills. There is little information on which knowledge items or skill steps are challenging for the learners.

**Methods:**

We used training data from NICHD's Global Network study to understand the items most challenging for Birth Attendants (BA) to guide future curriculum modifications. HBB training was provided in 15 primary, secondary and tertiary level care facilities in Nagpur, India. Refresher training was provided 6 months later. Each knowledge item and skill step was ranked from difficulty level 1 to 6 based on whether 91%–100%, 81%–90%, 71%–80%, 61%–70%, 51%–60% or <50% of learners answered/performed the step correctly.

**Results:**

The initial HBB training was conducted in 272 physicians and 516 midwives of which 78 (28%) physicians and 161 (31%) midwives received refresher training. Questions related to timing of cord clamping, management of a meconium-stained baby, and steps to improve ventilation were most difficult for both physicians and midwives. The initial steps of Objective Structured Clinical Examination (OSCE)-A i.e. equipment checking, removing wet linen and immediate skin-to-skin contact were most difficult for both groups. Midwives missed stimulating newborns while physicians missed cord clamping and communicating with mother. In OSCE-B, starting ventilation in the first minute of life was the most missed step after both initial and 6 months refresher training for physicians and midwives. At the retraining, the retention was worst for cutting the cord (physicians level 3), optimal rate of ventilation, improving ventilation & counting heart rate (midwives level 3), calling for help (both groups level 3) and scenario ending step of monitoring the baby and communicating with mother (physicians level 4, midwives 3).

**Conclusion:**

All BAs found skill testing more difficult than knowledge testing. The difficulty level was more for midwives than for physicians. So, the HBB training duration and frequency of retraining can be tailored accordingly. This study will also inform subsequent refinement in the curriculum so that both trainers and trainees will be able to achieve the required proficiency.

## Background

Approximately 3.3 million neonatal deaths occurred globally in 2009 (1), accounting for 41% of the deaths among under five years old (1). The vast majority of these deaths occur in low and middle-income countries (LMICs). India has 27.8% of global neonatal deaths and is among the five countries that make up more than half of all deaths (1). Most deaths take place within seven days of delivery (2, 3). A major cause of these deaths is asphyxia at birth (2). In high income countries (HIC) deliveries are usually attended by highly trained physicians/neonatologists or other medical staff who have adequate training to recognize an asphyxiated newborn and provide prompt resuscitation. In LMICs, even births that occur in medical facilities are not always attended by specialists or neonatologists. It is not uncommon that babies who are not breathing at birth are considered to be stillbirths and no efforts are made to provide resuscitation. Neonatal resuscitation trainings have thus helped to reduce perinatal mortality by reducing both the number of fresh stillbirths and early neonatal deaths (4, 5). A number of studies in past and recently published meta-analysis have evaluated the impact of essential newborn care and resuscitation training for physicians, nurses, and other birth attendants (BAs) on delivery outcome and neonatal survival (5). Many studies have also evaluated the impact of resuscitation trainings on the skills and knowledge of the trainees by assessing the changes in knowledge and skill scores before and after training (6, 7). Very few studies have assessed what knowledge items or skills steps are learning challenges for trainees i.e., what items the trainees are least likely to answer correctly during the resuscitation trainings or to retain over time. If the items that are most important for successful resuscitation are most difficult to master, then this failure to learn will impact survival of a newborn experiencing birth asphyxia. This information will also help guide the frequency of re-training to attain adequate proficiency in the items found most challenging for the trainees.

The HBB, is an evidence-based newborn resuscitation training program developed by a Global Development Alliance of five partners namely the American Academy of Pediatrics, the Eunice Kennedy Shriver National Institute of Child Health and Human Development (NICHD) Global Network, Save the Children, the Laerdal Global Health and the United States Agency for International Development (8, 9). It was developed to improve neonatal survival in low-resource settings by training first level birth attendants (BAs) to resuscitate neonates wherever they are born (10–12). The training module includes learning activities such as knowledge assessment using multiple choice questions, interactive scenario-based exercises, drills, simulation and role playing.

This program has now been implemented in several countries and has had an impact on resuscitation processes as well as neonatal survival (13, 14). However there is scope for improvement in order to accomplish the Every Newborn Action Plan goal of a global neonatal mortality rate of 7 per 1,000 live births by 2,035 (15). The NICHD's Global Network in collaboration with American Academy of Paediatrics and Laerdal Medical, conducted a study to evaluate the impact of implementation of HBB training and equipment package with continuous monitoring and quality improvement activities of the resuscitation practices on the perinatal mortality in Nagpur (India), Belgaum (India) and Eldoret (Kenya) (16, 17). During these trainings, pre-training and post-training tests were conducted to assess knowledge and skills of the trainees. At Nagpur site, training performance data was recorded for all the participants trained in HBB. This study aims to provide information regarding which knowledge items or skills steps were formidable for trainees' learning, thereby giving an insight into how the HBB training module can be further improved in terms of achieving its educational outcomes. It will allow policymakers, partners and trainers to adapt and improve the HBB training module curriculum with enhanced focus on areas which participants find difficult to learn. Thus, the objectives of this study were to determine, for birth attendants (physicians and midwives), the following - 1) the difficulty level of each knowledge and skill item of the HBB training package based on percent of trainees who could answer/perform it correctly, 2) to identify the items that were most responsive to HBB training, and, 3) to identify specific items for which the retention of knowledge and skills were lost over time.

## Methods

This study is secondary analysis of training performance data from a pre-post evaluation of a health facility-based training program in HBB at the Nagpur (India) site (16, 17). The HBB training program was implemented at 15 facilities (2 primary, 4 sary and 9 tertiary level care facilities) in and around Nagpur district, that provided round the clock delivery services (16). HBB curriculum and evaluation tools developed by American Academy of Pediatrics (AAP) for adult trainees of different backgrounds, with its high-quality teaching materials, were used according to their stated guidelines to train the BAs of the 15 participating facilities. The master trainers (MTs) were chosen carefully among doctors and nurses with adult education experience or an aptitude for teaching and expertise in neonatal resuscitation. These MTs first underwent a training of trainers (contact period of 24 h) where they had to give short demonstrations of their teaching and evaluation skills to ensure uniformity in teaching and evaluation thus preserving the integrity of the intervention (6).

The “initial” HBB training involved 14 contact hours of training of birth attendants by master trainers who were trained by AAP trainers (6, 16). The initial training had to include all facility staff, including the senior and administrative staff to increase the programmatic buy-in. Six months later, only those trainees from initial training that were actively engaged in attending births and resuscitating newborns (the “active” birth attendants- the health care workers that will be conducting deliveries in real world) received a “refresher” training of around five contact hours. There was no selection or sampling involved in this and ALL the “active” birth attendants received refresher training. BAs with previous training in neonatal resuscitation were not excluded. The details of the training including facility selection criteria, level of care these facilities provided and also the detailed characteristics of these active birth attendants have been previously published (6, 16). Between the initial and refresher trainings, the birth attendants underwent several monitoring activities best described as supportive supervision. These included daily practice of BMV skills at a well-equipped practice corner just before each duty shift with logbook entries; regular observation of deliveries by master trainers; debriefing after every resuscitation; and audits of every perinatal death. The current study assessed the knowledge and skill items included in the HBB curriculum that were challenging for the trainees.

### Evaluation of knowledge and skill levels

The detailed scheme of the trainings and evaluations is shown in Table 1. A multiple-choice questionnaire (MCQ) of 17 items (Questions) was used to assess “knowledge” of the participants. The knowledge assessment was done at the following time points - prior to the initial training to assess base-line knowledge prior to training. It was then conducted after initial training, prior to refresher training and after refresher training. Bag-Mask Ventilation (BMV) skills were assessed before and after the initial training using a checklist of 7 steps. Two Objective Structured Clinical Examinations (OSCE-A and OSCE-B) were used to evaluate the trainee's skills on the mannequin. OSCE-A had 13 steps and assessed ability of the trainee to resuscitate a newborn that required initial steps of resuscitation. OSCE-B assessed the resuscitation abilities during a more complex scenario of positive pressure ventilation through 18 steps. Individual questions and OSCE steps have been enumerated in Table 2. The OSCE-A and OSCE-B were not conducted prior to the initial training. They were conducted after the initial training. OSCE-B, which includes most skills of OSCE-A was also conducted before and after the refresher training that was conducted six months after the initial training. Based on the performance of participants during these trainings, we assessed the difficulty level of MCQs and skill steps included in BMV, OSCE-A and OSCE-B during the initial and refresher trainings. We also assessed which questions in the MCQs or skill steps of BMV, OSCE-A or OSCE-B were difficult to retain over a period of time. The pre–post training evaluation of trainees was conducted by master trainers and the responses of the all the MCQs and skill steps performed by the trainees were recorded on evaluation forms that provided the data for this study.

**Table 1:** Summary of pre - post training evaluation.

Evaluation	Initial Training		Refresher Training	
	Pre	Post	Pre	Post
Knowledge	MCQ	MCQ	MCQ	MCQ
Skill	BMV	BMV		BMV
		OSCE A		OSCE A
		OSCE B	OSCE B	OSCE B

MCQ, multiple-choice questionnaire; BMV, bag-mask ventilation; OSCE, objective structured clinical evaluation.

**Table 2:** List of questions and steps.

Knowledge Questions	Code	BMV Skills Steps	Code	OSCE B Skills Steps	Code
1. In the first minutes after birth, you should	MCQ1	1. Check equipment and select the correct mask	BMV01	1. Prepare for birth	OSB1
2. To prepare for a birth	MCQ2	2. Apply the mask to make to make a firm seal.	BMV02	2. Dries thoroughly and removes wet cloth	OSB2
3. To prepare the area for delivery	MCQ3	3. Ventilate at 40 breaths per minute.	BMV03	3. Evaluates crying Prompt: Recognizes baby is not crying	OSB3
4. Which baby can receive routine care after birth?	MCQ4	4. Look for chest movement.	BMV04	4. Evaluates crying Prompt: Keeps warm, positions head, clears airway	OSB4
5. Routine care for a healthy baby at birth	MCQ5	5. Improve ventilate if the chest does not move: Head - reapply mask and reposition head	BMV05	5. Evaluates crying Prompt: Stimulates breathing by rubbing the back	OSB5
6. When should the umbilical cord be clamped or tied and cut during routine care?	MCQ6	6. Improve ventilate if the chest does not move: Mouth - clear secretions and open the mouth	BMV06	6. Evaluates breathing: Recognizes baby is not breathing*	OSB6
7. A baby is quiet, limp & not breathing at birth. what should you do?	MCQ7	7. Improve ventilate if the chest does not move: Bag - squeeze the bag harder	BMV07	7. Cuts cord and moves to area for ventilation OR ventilates by mother	OSB7
8. A baby is born through meconium-stained amniotic fluid. Which statement is True?	MCQ8	OSCE A Skills Steps	Code	8. Starts ventilation within The Golden Minute SM (at seconds)	OSB8
9. What should you do in The Golden Minute Sm?	MCQ9	1. Identifies a helper and makes an emergency plan…	OSA1	9. Ventilates at 40 breaths/minute (30-50 acceptable) *	OSB9
10. A newborn baby is quiet, limp & not crying. The baby does not respond to steps to stimulate breathing. What should you do next?	MCQ10	2. Prepares the area for delivery…	OSA2	10. Looks for chest movement*	OSB10
11. Which of the following statements about ventilation with bag and mask is true?	MCQ11	3. Cleans hands and maintains clean technique throughout.	OSA3	11. Recognizes baby is not breathing	OSB11
12. Which of the following signs MUST be monitored in a baby during the first few hours after birth?	MCQ12	4. Prepares an area for ventilation and checks equipment.	OSA4	12. Calls for help	OSB12
13. A baby's chest is not moving with bag and mask ventilation. What should you do?	MCQ13	5. Dries thoroughly	OSA5	13. Continuous ventilation	OSB13
14. You can stop ventilation if	MCQ14	6. Removes wet cloth	OSA6	14. Improves ventilation*	OSB14
15. What should you do to keep the baby warm?	MCQ15	7. Recognizes baby is not crying	OSA7	15. Recognizes baby is not breathing but heart rate is normal	OSB15
16. What should you do to keep the baby clean?	MCQ16	8. Keeps warm	OSA8	16. Continues ventilation	OSB16
17. A newborn baby's heart rate should be:	MCQ17	9. Positions head and clears airways*	OSA9	17. Recognizes baby is breathing and heart rate is normal	OSB17
		10. Stimulates breathing by rubbing the baby	OSA10	18. Stops ventilation; monitors baby and communicates with mother	OSB18
		11. Recognizes baby is breathing well	OSA11		
		12. Clamps or ties and cuts the cord	OSA12		
		13. Positions skin-to-skin on mother's chest and communicates with mother	OSA13		

### Data analysis

The trainees or BAs were categorized into two groups – “Physicians” which included graduate and postgraduate doctors attending deliveries and the “Midwives” which included nurses, auxiliary nurse midwives (ANMs) and all other staff attending a delivery. Each MCQ and skill step was ranked from Level 1 to 6 difficulty level. These difficulty levels were based on the proportion of trainees who performed the knowledge or skill item correctly. The question or skill step that most trainees were able to do correctly was considered as the easiest or having the least level of difficulty. The difficulty levels were graded from 1 to 6 for passing percentage of 91%–100%, 81%–90%, 71%–80%, 61%–70%, 51%–60% and <50% respectively. For example, if an MCQ question was answered correctly by 91% of the learners, it was rated as difficulty Level 1. Although 100% proficiency is most desirable, for this study difficulty Level 1 was considered as an acceptable level of proficiency to assess improvement. If an MCQ question was answered correctly by only 49% of the learners, then it was rated as difficulty Level 6. We elected to use this method as it is intuitive and previously used for assessing difficulty levels of items (18).

### Ethical considerations

The study was reviewed and approved by the Institutional Review Board of Lata Medical Research Foundation, Nagpur. The ethics approval for the GN HBB study that trained the Nagpur site facilities in HBB have been published previously (6, 16, 17). Written informed consent was obtained from all trained birth attendants who participated in GN HBB study.

## Results

Total 788 trainees (272 physicians and 516 midwives) undertook the initial HBB training. Of these, all the 239 trainees (78 physicians and 161 midwives) who were actively involved as birth attendants received refresher training. Numbers and percentages of trainees passing in individual questions or steps with level wise color coding are provided in Tables 3A–D.

**Table 3A:** Percentage of trainees passing the individual knowledge assessment questions.

Question	Initial Training						Refresher Training					
	Pre			Post			Pre			Post		
	Physician	Midwives	Total	Physician	Midwives	Total	Physician	Midwives	Total	Physician	Midwives	Total
	# Pass (%)	# Pass (%)	# Pass (%)	# Pass (%)	# Pass (%)	# Pass (%)	# Pass (%)	# Pass (%)	# Pass (%)	# Pass (%)	# Pass (%)	# Pass (%)
	272	516	788	272	516	788	78	161	239	78	161	239
1. In the first minutes after birth, you should	264 (97)	467 (91)	731 (93)	265 (97)	500 (97)	765 (97)	74 (95)	154 (96)	228 (95)	77 (99)	160 (99)	237 (99)
2. To prepare for a birth	259 (95)	382 (74)	641 (81)	268 (99)	483 (94)	751 (95)	72 (92)	149 (93)	221 (92)	76 (97)	156 (97)	232 (97)
3. To prepare the area for delivery	269 (99)	487 (94)	756 (96)	271 (100)	502 (97)	773 (98)	77 (99)	155 (96)	232 (97)	78 (100)	157 (98)	235 (98)
4. Which baby can receive routine care after birth?	252 (93)	453 (88)	705 (89)	267 (98)	498 (97)	765 (97)	76 (97)	148 (92)	224 (94)	76 (97)	151 (94)	227 (95)
5. Routine care for a healthy baby at birth	259 (95)	404 (78)	663 (84)	270 (99)	490 (95)	760 (96)	77 (99)	148 (92)	225 (94)	77 (99)	151 (94)	228 (95)
6. When should the umbilical cord be clamped or tied and cut during routine care?	169 (62)	286 (55)	455 (58)	265 (97)	485 (94)	750 (95)	67 (86)	137 (85)	204 (85)	72 (92)	153 (95)	225 (94)
7. A baby is quiet, limp& not breathing at birth. What should you do	207 (76)	270 (52)	477 (61)	262 (96)	447 (87)	709 (90)	75 (96)	137 (85)	212 (89)	75 (96)	151 (94)	226 (95)
8. A baby is born through meconium-stained amniotic fluid. Which statement is True?	228 (84)	280 (54)	508 (64)	257 (94)	417 (81)	674 (86)	68 (87)	88 (55)	156 (65)	69 (88)	114 (71)	183 (77)
9. What should you do in The Golden Minute^Sm^?	237 (87)	389 (75)	626 (79)	266 (98)	471 (91)	737 (94)	68 (87)	133 (83)	201 (84)	71 (91)	146 (91)	217 (91)
10. A newborn baby is quiet, limp& not crying. The baby does not respond to steps to stimulate breathing. What should you do next?	227 (83)	273 (53)	500 (63)	268 (99)	478 (93)	746 (95)	72 (92)	139 (86)	211 (88)	77 (99)	152 (94)	229 (96)
11. Which of the following statements about ventilation with bag and mask is true?	191 (70)	257 (50)	448 (57)	235 (86)	391 (76)	626 (79)	58 (74)	94 (58)	152 (64)	71 (91)	134 (83)	205 (86)
12. Which of the following signs MUST be monitored in a baby during the first few hours after birth?	256 (94)	450 (87)	706 (90)	266 (98)	499 (97)	765 (97)	75 (96)	153 (95)	228 (95)	74 (95)	153 (95)	227 (95)
13. A baby's chest is not moving with bag and mask ventilation. What should you do?	255 (94)	326 (63)	581 (74)	269 (99)	465 (90)	734 (93)	71 (91)	141 (88)	212 (89)	76 (97)	150 (93)	226 (95)
14. You can stop ventilation if	256 (94)	404 (78)	660 (84)	262 (96)	468 (91)	730 (93)	68 (87)	135 (84)	203 (85)	73 (94)	153 (95)	226 (95)
15. What should you do to keep the baby warm?	264 (97)	460 (89)	724 (92)	271 (100)	498 (97)	769 (98)	75 (96)	151 (94)	226 (95)	78 (100)	156 (97)	234 (98)
16. What should you do to keep the baby clean?	266 (98)	450 (87)	716 (91)	269 (99)	495 (96)	764 (97)	74 (95)	154 (96)	228 (95)	78 (100)	156 (97)	234 (98)
17. A newborn baby's heart rate should be:	269 (99)	482 (93)	751 (95)	270 (99)	501 (97)	771 (98)	76 (97)	151 (94)	227 (95)	77 (99)	158 (98)	235 (98)

**Table 3B:** Percentage of trainees passing the individual Bag-mask ventilation steps.

Question	Initial Training						Refresher Training					
	Pre			Post			Pre			Post		
	Physician	Midwives	Total	Physician	Midwives	Total	Physician	Midwives	Total	Physician	Midwives	Total
	# Pass (%)	# Pass (%)	# Pass (%)	# Pass (%)	# Pass (%)	# Pass (%)	# Pass (%)	# Pass (%)	# Pass (%)	# Pass (%)	# Pass (%)	# Pass (%)
	272	516	788	272	516	788	78	161	239	78	161	239
1. Check equipment and select the correct mask	106 (39)	154 (30)	260 (33)	256 (94)	504 (98)	760 (96)				75 (96)	152 (94)	227 (95)
2. Apply the mask to make to make a firm seal.	170 (63)	171 (33)	341 (43)	265 (97)	496 (96)	761 (97)				75 (96)	151 (94)	226 (95)
3. Ventilate at 40 breaths per minute.	106 (39)	92 (18)	198 (25)	252 (93)	446 (86)	698 (89)				74 (95)	151 (94)	225 (94)
4. Look for chest movement.	151 (56)	154 (30)	305 (39)	261 (96)	456 (88)	717 (91)				73 (94)	150 (93)	223 (93)
5. Improve ventilate if the chest does not move: Head - reapply mask and reposition head	106 (39)	68 (13)	174 (22)	265 (97)	480 (93)	745 (95)				75 (96)	154 (96)	229 (96)
6. Improve ventilate if the chest does not move Mouth - clear secretions and open the mouth	58 (21)	48 (9)	106 (13)	262 (96)	475 (92)	737 (94)				72 (92)	151 (94)	223 (93)
7. Improve ventilate if the chest does not move: Bag - squeeze the bag harder	61 (22)	42 (8)	103 (13)	223 (82)	388 (75)	611 (78)				73 (94)	149 (93)	222 (93)

**Table 3C:** Percentage of trainees passing the individual OSCE – A steps.

Question	Initial Training						Refresher Training					
	Pre			Post			Pre			Post		
	Physician	Midwives	Total	Physician	Midwives	Total	Physician	Midwives	Total	Physician	Midwives	Total
	# Pass (%)	# Pass (%)	# Pass (%)	# Pass (%)	# Pass (%)	# Pass (%)	# Pass (%)	# Pass (%)	# Pass (%)	# Pass (%)	# Pass (%)	# Pass (%)
	272	516	788	272	516	788	78	161	239	78	161	239
1. Identifies a helper and makes an emergency plan…				267 (98)	494 (96)	761 (97)				75 (96)	154 (96)	229 (96)
2. Prepares the area for delivery…				261 (96)	486 (94)	747 (95)				75 (96)	150 (93)	225 (94)
3. Cleans hands and maintains clean technique throughout.				257 (94)	472 (91)	729 (93)				74 (95)	151 (94)	225 (94)
4. Prepares an area for ventilation and checks equipment.				247 (91)	459 (89)	706 (90)				73 (94)	151 (94)	224 (94)
5. Dries thoroughly				265 (97)	490 (95)	755 (96)				75 (96)	154 (96)	229 (96)
6. Removes wet cloth				247 (91)	448 (87)	695 (88)				68 (87)	145 (90)	213 (89)
7. Recognizes baby is not crying				267 (98)	484 (94)	751 (95)				75 (96)	151 (94)	226 (95)
8. Keeps warm				248 (91)	469 (91)	717 (91)				72 (92)	146 (91)	218 (91)
9. Positions head and clears airways*				267 (98)	482 (93)	749 (95)				74 (95)	152 (94)	226 (95)
10. Stimulates breathing by rubbing the baby				257 (94)	457 (89)	714 (91)				70 (90)	145 (90)	215 (90)
11. Recognizes baby is breathing well				260 (96)	469 (91)	729 (93)				73 (94)	151 (94)	224 (94)
12. Clamps or ties and cuts the cord				262 (96)	479 (93)	741 (94)				69 (88)	149 (93)	218 (91)
13. Positions skin-to-skin on mother's chest and communicates with mother				247 (91)	462 (90)	709 (90)				65 (83)	146 (91)	211 (88)

**Table 3D:** Percentage of trainees passing the individual OSCE – B steps.

Question	Initial Training						Refresher Training					
	Pre			Post			Pre			Post		
	Physician	Midwives	Total	Physician	Midwives	Total	Physician	Midwives	Total	Physician	Midwives	Total
	# Pass (%)	# Pass (%)	# Pass (%)	# Pass (%)	# Pass (%)	# Pass (%)	# Pass (%)	# Pass (%)	# Pass (%)	# Pass (%)	# Pass (%)	# Pass (%)
	272	516	788	272	516	788	78	161	239	78	161	239
1. Prepare for birth				263 (97)	476 (92)	739 (94)	70 (90)	141 (88)	211 (88)	74 (95)	153 (95)	227 (95)
2. Dries thoroughly and removes wet cloth				265 (97)	481 (93)	746 (95)	69 (88)	135 (84)	204 (85)	76 (97)	149 (93)	225 (94)
3. Evaluates crying Prompt: Recognizes baby is not crying				244 (90)	447 (87)	691 (88)	73 (94)	140 (87)	213 (89)	76 (97)	153 (95)	229 (96)
4. Evaluates crying Prompt: Keeps warm, positions head, clears airway				268 (99)	476 (92)	744 (94)	68 (87)	139 (86)	207 (87)	74 (95)	148 (92)	222 (93)
5. Evaluates crying Prompt: Stimulates breathing by rubbing the back				240 (88)	440 (85)	680 (86)	70 (90)	135 (84)	205 (86)	74 (95)	147 (91)	221 (92)
6. Evaluates breathing: Recognizes baby is not breathing*				255 (94)	481 (93)	736 (93)	71 (91)	141 (88)	212 (89)	73 (94)	146 (91)	219 (92)
7. Cuts cord and moves to area for ventilation OR ventilates by mother				247 (91)	457 (89)	704 (89)	61 (78)	132 (82)	193 (81)	71 (91)	147 (91)	218 (91)
8. Starts ventilation within The Golden Minute SM (at seconds)				228 (84)	344 (67)	572 (73)	51 (65)	107 (66)	158 (66)	67 (86)	126 (78)	193 (81)
9. Ventilates at 40 breaths/minute (30–50 acceptable)*				262 (96)	474 (92)	736 (93)	65 (83)	121 (75)	186 (78)	74 (95)	147 (91)	221 (92)
10. Looks for chest movement*				262 (96)	482 (93)	744 (94)	69 (88)	134 (83)	203 (85)	75 (96)	149 (93)	224 (94)
11. Recognizes baby is not breathing				257 (94)	462 (90)	719 (91)	70 (90)	137 (85)	207 (87)	75 (96)	150 (93)	225 (94)
12. Calls for help				240 (88)	413 (80)	653 (83)	58 (74)	120 (75)	178 (74)	70 (90)	136 (84)	206 (86)
13. Continuous ventilation				265 (97)	470 (91)	735 (93)	69 (88)	139 (86)	208 (87)	74 (95)	147 (91)	221 (92)
14. Improves ventilation*				263 (97)	475 (92)	738 (94)	69 (88)	126 (78)	195 (82)	74 (95)	149 (93)	223 (93)
15. Recognizes baby is not breathing but heart rate is normal				255 (94)	452 (88)	707 (90)	68 (87)	127 (79)	195 (82)	70 (90)	147 (91)	217 (91)
16. Continues ventilation				261 (96)	475 (92)	736 (93)	69 (88)	138 (86)	207 (87)	75 (96)	147 (91)	222 (93)
17. Recognizes baby is breathing and heart rate is normal				252 (93)	467 (91)	719 (91)	53 (68)	132 (82)	185 (77)	69 (88)	146 (91)	215 (90)
18. Stops ventilation; monitors baby and communicates with mother				241 (89)	442 (86)	683 (87)	54 (69)	127 (79)	181 (76)	73 (94)	141 (88)	214 (90)

### Knowledge assessment – 17 MCQs

In knowledge assessment, at baseline (prior to initial training), for physicians, out of total 17 questions, two questions were graded as Level 4 difficulty (Questions 6, 11) and three were graded as Level 2 (Questions 8, 9, 10) difficulty (Table 3A). The difficulty levels of all questions improved to Level 1 after training with the exception of Question 11 that improved from Level 4 to Level 2. After 6 months, the difficulty level of Question 11 was at Level 3 and for four questions (Questions 6, 8, 9, 14) it was at Level 2 difficulty as compared to difficulty Level 1 observed after the initial training.

At base-line (prior to initial training) midwives found all questions to be above difficulty Level 1 with the exception of three questions i.e., Question 1, Question 3 and Question 17. Of the fourteen questions above difficulty Level 1, one question was of difficulty Level 6 (Question 11), four at Level 5, one at Level 4, four at Level 3 and four at Level 2. After training, four questions remained to be difficult, of these, three questions (Questions 7, 8 and 13) at difficulty Level 2 and Question 11 improved from Level 6 to Level 3. After 6 months, before the refresher training, the midwives found Question 8 and 11 to be of difficulty Level 5 and six other questions (Question 6, 7, 9, 10, 13, 14) at difficulty Level 2. After the refresher training, only two questions remained above difficulty Level 1 i.e., Question 8 at difficulty Level 3 and Question 11 at difficulty Level 2.

### Bag & mask ventilation - 7 skill steps

BMV skill assessment showed that both physicians and midwives found all steps to be of difficulty Level 6 before the initial training (Table 3B). After training, the physicians found only Step 7 to be of difficulty Level 2. The midwives found Step 3 and 4 to be of difficulty Level 2 and Step 7 was at difficulty Level 3. After refresher training, all trainees found the steps to be in the range of difficulty Level 1.

### OSCE-A - 13 skill steps

OSCE-A test was conducted after the initial training (Table 1). The physicians performed well in all the steps during this training (Table 3C). After refresher training the physicians faltered on Step 6 on removal of the wet cloth, Step 10 of stimulation of breathing by rubbing the baby, Step 12 regarding cord clamping and Step13 regarding skin-to-skin positioning and communicate with mother (difficulty Level 2). After the initial training, the midwives faltered on Step 4 in preparation of area for ventilation and equipment check, Step 6 on removal of the wet cloth, Step 10 of stimulation of breathing by rubbing the baby and Step 13 on skin-to-skin positioning and communicate with mother. These steps were in the range of difficulty Level 2. After refresher training, midwives again faltered (difficulty Level 2) on Question 6 of removal of wet cloth and Step 10 regarding stimulation of breathing by rubbing the baby.

### OSCE-B - 18 skill steps

OSCE-B skill evaluation was conducted after initial training, before refresher, and, after refresher training (Table 1). After initial training, the physicians found five steps (Step 3 recognizing baby not crying, Step 5 stimulation of breathing by rubbing the back, Step 8 on starting the ventilation within Golden Minute, Step 12 call for help and Step 18 monitoring the baby and communicate with mother) to be in the range of difficulty Level 2 (Table 3D). The remaining thirteen steps were within the range of difficulty Level 1. Prior to the refresher training, the evaluation showed that the physicians faltered on all steps (difficult levels ranging from Level 2 to 4) except Step 3 and Step 6 on recognition of failure to cry and failure to breath respectively which were at difficulty Level 1. Step 8 of starting ventilation in Golden Minute, Step 17 of recognition that baby continues to breath and have normal heart rate, and, Step 18 of stopping ventilation, monitoring and communicating with mother were at difficulty Level 4. After the refresher training the performance for Step 8 on starting ventilation in Golden Minute, Step 12 on Call for help, Step 15 on recognition that baby is not breathing but heart rate is normal and Step 17 recognition that baby continues to breath and normal heart rate, failed to reach difficult Level 1 and were at Level 2.

After initial training, the midwives found eight steps difficult - Step 3 on recognition of failure to cry, Step 5 stimulation of breathing by rubbing the back, Step 7 cut the cord and ventilate, Step 8 starting the ventilation within Golden Minute, Step 11 recognition that baby is not breathing, Step 12 call for help, Step 15 recognition that baby in not breathing but heart rate is normal and Step 18 monitoring the baby and communicate with mother. The difficulty level was in the range of Level 2 to 4. Prior to the refresher training, the evaluation showed that the midwives faltered on all the steps (difficult levels ranging from Level 2 to 4).

After the refresher training the performance for Step 8 - starting ventilation in Golden Minute was at difficulty Level 3 whereas Step 12 call for help and Step 18 monitoring the baby and communicate with mother were at level 2.

The summary of the difficulty level grading of individual steps at various time points is presented in the Tables 4A–C.

**Table 4A:** Difficulty level grading of individual knowledge assessment questions.

Knowledge Evaluation Using Multiple Choice Questions						
Difficulty Level	Physician			Midwives		
	Baseline Pre-Initial	Post-Initial	Retention Pre-Refresher	Pre-Initial	Post-Initial	Retention Pre-Refresher
Level 1 (91–100)	MCQ1, MCQ2, MCQ3, MCQ4, MCQ5, MCQ12, MCQ13, MCQ14, MCQ15, MCQ16, MCQ17	MCQ1, MCQ2, MCQ3, MCQ4, MCQ5, MCQ6, MCQ7, MCQ8, MCQ9, MCQ10, MCQ12, MCQ13, MCQ14, MCQ15, MCQ16, MCQ17	MCQ1, MCQ2, MCQ3, MCQ4, MCQ5, MCQ7, MCQ10, MCQ12, MCQ13, MCQ15, MCQ16, MCQ17	MCQ1, MCQ3, MCQ17	MCQ1, MCQ2, MCQ3, MCQ4, MCQ5, MCQ6, MCQ9, MCQ10, MCQ12, MCQ14, MCQ15, MCQ16, MCQ17	MCQ1, MCQ2, MC3, MCQ4, MCQ05, MCQ12, MCQ15, MCQ16, MCQ17
Level 2 (81–90)	MCQ8, MCQ9, MCQ10	MCQ11	MCQ6, MCQ8, MCQ9, MCQ14	MCQ4, MCQ12, MCQ15, MCQ16	MCQ7, MCQ8, MCQ13	MCQ6, MCQ7, MCQ9, MCQ10, MCQ13, MCQ14
Level 3 (71–80)			MCQ11	MCQ2, MCQ5, MCQ9, MCQ14,	MCQ11	
Level 4 (61–70)	MCQ6, MCQ11			MCQ13		
Level 5 (51–60)				MCQ6, MCQ7, MCQ8, MCQ10		MCQ8, MCQ11
Level 6 (50 or below)				MCQ11		

**Table 4B:** Difficulty level grading of individual Bag mask ventilation steps.

Skills Evaluation Using Bag Mask Ventilation						
Difficulty Level	Physician			Midwives		
	Baseline Pre Initial	Post Initial	Retention Pre Refresher	Pre Initial	Post Initial	Retention Pre Refresher
Level 1 (91–100)		BMV1, BMV2, BMV3, BMV4, BMV5, BMV6			BMV1, BMV2, BMV5, BMV6	
Level 2 (81–90)		BMV7			BMV3, BMV4	
Level 3 (71–80)					BMV7	
Level 4 (61–70)	BMV2					
Level 5 (51–60)	BMV4					
Level 6 (50 or below)	BMV1, BMV3, BMV5, BMV6, BMV7			BMV1, BMV2, BMV3, BMV4, BMV5, BMV6, BMV7		

**Table 4C:** Difficulty level grading of individual OSCE-B steps.

Skills Evaluation Using OSCE B						
Difficulty Level	Physician			Midwives		
	Baseline Pre Initial	Post Initial	Retention Pre Refresher	Pre Initial	Post Initial	Retention Pre Refresher
Level 1 (91–100)		OSB1, OSB2, OSB4, OSB6, OSB7, OSB9, OSB10, OSB11, OSB13, OSB14, OSB15, OSB16, OSB17	OSB3, OSB6		OSB1, OSB2, OSB4, OSB6, OSB9, OSB10, OSB13, OSB14, OSB16, OSB17	
Level 2 (81–90)		OSB03, OSB05, OSB08, OSB12, OSB18	OSB1, OSB2, OSB4, OSB5, OSB9, OSB10, OSB11, OSB13, OSB14, OSB15, OSB16		OSB3, OSB5, OSB7, OSB11, OSB15, OSB18	OSB1, OSB2, OSB3, OSB4, OSB5, OSB6, OSB7, OSB10, OSB11, OSB13, OSB16, OSB17
Level 3 (71–80)			OSB7, OSB12		OSB12	OSB9, OSB12, OSB14, OSB15, OSB18
Level 4 (61–70)			OSB8, OSB17, OSB18		OSB8	OSB8
Level 5 (51–60)						
Level 6 (50 or below)						

## Discussion

This study was conducted at one of the sites of a large multi-national pre-post study to evaluate the impact of HBB training at facilities on neonatal survival in the communities. In the educational outcomes evaluation, we assessed which knowledge items and skill steps need greater attention and emphasis during training so that optimal performance can be achieved by the trainees. To the best of our knowledge there are few studies world- wide that have conducted an itemized analysis of MCQs or OSCEs used in the HBB training for both physician and nurse learners. This analysis was conducted for the first time in Indian trainees to identify specific deficits in knowledge and performance that can be targeted for improvement and to help strengthen the HBB curriculum. It was observed that both types of BAs found skill testing such as the BMV, OSCE-A and OSCE-B to be more challenging than responding to MCQs. These findings concur with previous studies of resuscitation training that report low concordance between knowledge and skills (10, 19). It has been noted that acquisition of knowledge occurred more easily than skills (20). The physician trainees found both knowledge items and the skill step less difficult to learn as compared to midwives. The same knowledge questions and skill steps that physicians found difficult were also found difficult by the midwives but the degree of difficulty was more for the latter. This was also observed after six months of initial training when knowledge or skill retention was poorer for midwives as compared to physicians and similar questions and steps were found to be difficult (6). Similar findings were observed in the study conducted in Honduras where seventy physicians and nurses participated in the HBB training. It is known that learners arrive with varied prior knowledge and skills. These consistent findings could inform future HBB trainings. It was evident that physicians differed both in awareness of learning strategies and pace of learning compared to the nurses. The program could consider tailoring the training based on learner characteristics and background (21). The physicians and nurses may need to be trained separately as the time to learn can be different. The frequency of subsequent retraining for nurses and physicians could also differ. There could also be a role for incorporating learner-adaptive educational technologies for HBB training that use software and hardware systems that deliver interactive, individualized content by accruing data from a learner to infer what is not understood by the learner so that the simulation models can address those learning needs. Adaptive learning technologies can provide interactive, individualized content that can be delivered at scale. This is particularly relevant for the health personnel who can then train at their convenient times when not on duty.

Among the MCQs, the question that was found to be most difficult and would need attention during future trainings was Question 11 (true of false statements regarding BMV). The midwives also found Question 6 to 14 pertaining to when to clamp cord, recognition of limp baby, response to limp and not breathing baby, steps to be taken for meconium-stained amniotic fluid, what should be done in the Golden Minute, steps to be taken when chest does not move with BMV and when to stop ventilation to be difficult. It was evident that BAs did not perform well in knowledge questions that pertain to resuscitation skills. The trainees need to be well versed with the learner's manual prior to the workshop and the trainers need to explain the resuscitation steps well and carefully using the flip charts.

The MCQs assess clinical knowledge but the OSCEs help evaluate procedural skills, clinical decision making, communication etc during the resuscitation process. OSCEs are considered as standard assessment tools for clinical competence (22). OSCE-A (13 steps) was tested after initial and after refresher training. The midwives who are usually responsible for preparing the area of ventilation and checking equipment were the ones who faltered on it indicating that during routine clinical practice these steps were perhaps not being followed meticulously. Therefore, trainers should not presume that active midwives are routinely practicing these preparatory steps and provide adequate time and attention to teach these steps. The trainees were also not proficient in removal of wet cloth, stimulation of breathing by rubbing the baby, positioning the baby on mother's chest and communicating with her. Again, the trainers should not rely on the clinical experience of midwives and give sufficient attention to these steps for achieving optimum performance among the trainees as removing the wet cloth is important to prevent hypothermia in the newborn whereas stimulation of breathing by rubbing the baby is often enough to establish respiration and avoid BMV (23). We have previously reported the knowledge and skill gaps amongst the BAs and improvement subsequent to trainings (6). We concluded that skills declined more than knowledge over time and that in addition to frequent refresher training the BAs need to practice daily at practice corners established at their clinical sites.

OSCE-B evaluated the comprehensive skill that included the skills and decision-making in routine care, the initial steps of “The Golden Minute”, bag-mask ventilation and additional decision making based on assessment of heart rate after BMV. Most of the BMV steps and OSCE-A steps are incorporated in 18 steps of OSCE-B. The trainees faltered on most of the steps to establish ventilation and even after establishing it they would falter on how to continue to monitor breathing, monitor heart rate and finally how to communicate with the mother. It is possible that the trainees tend to relax after breathing is established and are less attentive towards subsequent monitoring, when to stop and communicate with the mother. This indicates that trainers need to emphasize on all the steps from preparation to establishment of breathing, subsequent stabilization and final communication with the mother for optimum newborn survival. Seto et al. also conducted an itemized analysis of OSCEs. Despite a different site and study population the study did not yield different results from our study. They found similar steps in both the OSCEs to be challenging or have good item discrimination (ability to differentiate between low and high performers) (21). The authors recommended simplification of multi-steps into single tasks, use of global rating scale, discussions on scenarios, more self-reflection to enhance de-briefings and feedbacks. Poor performance in the OSCEs could possibly be attributed to lack of familiarity with the OSCE format or checklists prior to training. Learners may not have sufficient time to practice and there could be difficulty in remembering and integrating the large number of steps into a continuous series resulting in poor performance. Facilitators also need to be experienced and familiarize themselves well with the structured curriculum to teach it effectively (10).

A study done by the architect of HBB at Kenya and Pakistan conducted a formative evaluation of HBB program which included the course evaluation by facilitators and learners using quantitative ratings and qualitative feedback through comments and focus group discussion on structure, course content, materials and assessment tools. They assessed the participants knowledge, skills and performance but did not analyze the individual question or skill steps that were difficult for trainees to perform (10). The participants expressed satisfaction with the program and the knowledge and skills pre/post-program demonstrated significant gains; however, in that study too, majority of participants could not demonstrate mastery of BMV perhaps due to insufficient practice. The participants cited limited time to practice the integration of separate skills. The formative evaluation helped further revise the course materials by including larger illustrations, integrating the skill practice into the presentation of knowledge content, provide additional exercises with guided instructions and provided for increased time to practice and consolidate the skills. Despite the revision in the curriculum, the trainees of our study faltered in both the knowledge and the skill domain.

The strength of this study is that it provides an itemized assessment of the difficulty level of the curriculum to help future trainings. The limitation is that it could be both site and learner specific. The apparently smaller number at the refresher training was due to exclusion of administrative and senior staff who never attended deliveries. All the active birth attendants received refresher training without any sampling to avoid selection bias. Since these are the health care workers who would be attending the deliveries in the real world, our results can be generalized to all birth attendants in similar settings. Standard, uniform training curriculum; carefully maintained integrity of training and evaluation by ensuring uniformity across trainers; and detailed enlisting of monitoring activities between the initial and refresher trainings are the other strengths of our study.

In conclusion, this study has shown that health care workers find certain skills more difficult to learn and retain. HBB training duration and frequency of retraining can be tailored accordingly with greater emphasis on acquisition of these skills during trainings and during the ongoing practice and monitoring. This study will also inform subsequent refinement in the curriculum so that both trainers and trainees will be able to achieve the required proficiency. Further evaluation and additional learner/facilitator assessment data from diverse settings and study populations will help to inform subsequent refinements to the HBB curriculum.

## Data Availability

The original contributions presented in the study are included in the article/Supplementary Material, further inquiries can be directed to the corresponding author.
